# Multimarker RT–PCR assay for the detection of minimal residual disease in sentinel lymph nodes of breast cancer patients

**DOI:** 10.1038/sj.bjc.6602992

**Published:** 2006-02-21

**Authors:** A Nissan, D Jager, M Roystacher, D Prus, T Peretz, I Eisenberg, H R Freund, M Scanlan, G Ritter, L J Old, S Mitrani-Rosenbaum

**Affiliations:** 1 Department of Surgery and Surgical Oncology Laboratory, Hadassah-Hebrew University Medical Center, Jerusalem, Israel; 2 Klinische Onkologie im NCT, Universitätsklinikum Heidelberg, Heidelberg, Germany; 3 Department of Pathology, Hadassah-Hebrew University Medical Center, Jerusalem, Israel; 4 Department of Oncology, Hadassah-Hebrew University Medical Center, Jerusalem, Israel; 5 The Ludwig Institute for Cancer Research, New York Branch, New York, NY, USA; 6 The Goldyne Savad Institute of Gene Therapy, Hadassah-Hebrew University Medical Center, Jerusalem, Israel

**Keywords:** RT–PCR, breast neoplasms, micrometastases, mammaglobin, NY-BR-1

## Abstract

The presence of metastases in lymph nodes is the most powerful prognostic factor in breast cancer patients. Routine histological examination of lymph nodes has limited sensitivity for the detection of breast cancer metastases. The aim of the present study was to develop a multimarker reverse transcriptase–polymerase chain reaction (RT—PCR) assay for the detection of minimal residual disease in sentinel nodes of breast cancer patients. RNA was extracted from 30 sentinel lymph nodes (SLN) obtained from 28 patients, three primary breast cancers (positive controls), three lymph nodes from patients with benign diseases, and peripheral blood lymphocytes of 10 healthy volunteers (negative controls). RT–PCR was performed using the following markers; cytokeratin (CK)-19, NY-BR-1 and mammaglobin B. RT–PCR results were compared to enhanced histopathologic examination and immunohistochemistry (IHC). All three positive controls showed strong PCR amplification for all three markers. None of the 13 negative controls was amplified by any of the three markers. Among the 30 SLN analysed, breast cancer metastases were detected in six SLNs by routine histology, in eight by IHC and in 15 by RT–PCR. We conclude that a multimarker RT–PCR assay probing for NY-BR-1, mammaglobin-B, and CK-19 is more sensitive compared to enhanced pathologic examination. This method may prove to be of value in breast cancer staging and prognosis evaluation.

Although up to 80% of breast cancer patients are identified at an early stage when disease is apparently localised, 30–35% of node-negative patients will develop clinically detectable distant metastases within 5 years of treatment of the primary breast carcinoma and ultimately suffer tumour-related mortality (Nemoto *et al*, 1980). Routine histological examination of lymph nodes has limited sensitivity for the detection of breast cancer metastases. It is usually performed with a single haematoxilyn and eosin (H&E)-stained section through the examined node and therefore small metastases can go undetected. In a landmark study conducted by the International (Ludwig) Breast Cancer Group (International (Ludwig) Breast Cancer Study Group, 1990), serial sectioning of axillary lymph nodes judged to be disease free after routine histological examination revealed micrometastases in 83 (9%) of 921 breast cancer patients. These patients had a poorer disease-free (*P*=0.003) and overall (*P*=0.002) survival after 5 years’ median follow-up than did patients whose nodes remained negative on serial sectioning. A later study performed by the same group (Cote *et al*, 1999) examined lymph nodes negative for metastases on routine histology from 736 patients by serial sectioning and by immunohistochemistry (IHC) with two anticytokeratins AE-1 and CAM 5.2. Occult nodal metastases were detected by serial sectioning and H&E in 7% of the patients and by IHC in 20%. After median follow-up of 12 years, occult metastases, detected by either method, were associated with significantly poor disease-free and overall survival in postmenopausal but not in premenopausal patients. Other studies have also shown that breast cancer micrometastases in axillary lymph nodes being detected by serial sectioning and IHC have prognostic significance (Braun *et al*, 2000, 2001). Reverse transcriptase–polymerase chain reaction (RT–PCR) has a potential of increasing the sensitivity of the pathologic processing of lymph nodes obtained from breast cancer patients. However, the specificity of RT–PCR is lower as compared to IHC and the prognostic significance of breast cancer micrometastases detected by RT–PCR is yet to be determined. Studies in malignant melanoma patients showed that detection of mRNA of the enzyme tyrosinase by RT–PCR reaction in sentinel lymph nodes (SLN) correlates with disease recurrence and survival (Shivers *et al*, 1998; Li *et al*, 2000). Several studies utilised mRNA detection by RT–PCR to detect minimal residual disease (MRD) in breast cancer patients (Schoenfeld *et al*, 1994; Noguchi *et al*, 1996; Aihara *et al*, 1999; Inokuchi *et al*, 2003). Different mRNA markers and RT–PCR techniques were used for the detection of breast cancer micrometastases and therefore results vary between investigators. Epithelial nonspecific markers such as cytokeratin (CK)-19 or CK-20 are present on every breast cancer cell but the risk of contamination by noncancer epithelial cells during surgery exists. Small amounts of CK were reported to be expressed in interstitial reticulum cells present in normal lymph nodes (Gould *et al*, 1995). Using epithelial mRNA markers carries a risk of false-positive results. Cancer-related mRNA markers are more specific but are not uniformly expressed in all tumours and therefore the risk of false-negative results exists. Thus, a combination of both epithelial and cancer-specific mRNA markers could potentially yield the most reliable results.

However, enhanced pathologic examination of multiple lymph nodes requires substantial resources and is time consuming. Therefore, sentinel lymph node biopsy which replaced routine axillary lymph node dissection (ALND) in breast cancer patients allows the pathologist to examine one or two lymph nodes in more detail as compared to an ALND specimen containing multiple lymph nodes. The ability of this surgical technique to locate the first lymph node that drains the tumour and therefore most likely to harbor occult metastases is of great value for the detection of micrometastatic disease.

The aim of the present study was develop an accurate multimarker RT–PCR assay for the detection of MRD in sentinel nodes of breast cancer patients. We elected to use two specific mRNA markers mammaglobin-B (MGB-2 and NY-BR-1) highly expressed in breast cancer (Becker *et al*, 1998; Jager *et al*, 2001) combined with CK-19.

## PATIENTS AND METHODS

Twenty-eight patients with histologically confirmed primary adenocarcinoma of the breast underwent sentinel lymphadenectomy performed by a single attending surgeon at Hadassah-Hebrew University Hospital Mount Scopus. Patients with clinical lymph node involvement or distant metastases were excluded from the study.

### Operative technique

Unfiltered technetium Tc 99m sulphur colloid was injected around the primary lesion or biopsy site 2–24 h before surgery. Preoperative lymphoscintigraphy was performed prior to surgery, once the sentinel node was visualised, the patients were transferred to the operating room. Vital blue dye was injected at the site of the primary tumour. Using a handheld gamma probe (Neoprobe 2000®, Neoprobe Inc., Dublin, OH, USA), the sentinel lymph node was localised identified and excised.

### Sentinel lymph node processing

In all, 30 SLN specimens were submitted fresh from the operating room in a sterile fashion. Each SLN was bivalved, one-half of each specimen was formalin fixed and paraffin embedded for standard pathologic examination, and the remainder of the SLN was snap frozen in liquid nitrogen for subsequent molecular analysis by RT–PCR. Multiple formalin-fixed, paraffin-embedded, H&E-stained sections were examined. Immunohistochemistry stains with two anticytokeratin antibodies AE-1 and CAM 5.2 were performed.

### RNA extraction

Frozen samples were powdered in dry ice, homogenised using a Polytron homogeniser in TriReagent (Molecular Research Center Inc., Cincinnati, OH, USA) and processed according to the manufacturer's instructions.

### Reverse transcription–polymerase chain reaction

Reverse transcription was performed on 1–5 *μ*g RNA, using Superscript II RNase H-Reverse Transcriptase (Invitrogen, Carlsbad, CA, USA) and random hexamer primers (Roche, Mannheim, Germany).

Subsequently, PCR was performed on 1/10th of the cDNA reaction volume with the corresponding primers and at the appropriate conditions.

### Oligonucleotide primers and probes

Oligonucleotide primers and internal probes were synthesised at MWG-Biotech AG (Ebersberg, Germany). Each set of primers specifically amplified and detected, respectively, a 462 bp fragment of the CK-19 gene, a 245 bp fragment of mammaglobin-B (MGB-2) and a 573 bp fragment of NY-BR-1. The sequence of these oligonucleotides is as follows:

CK-19 (corresponding gene is *KRT19*):

CK-19F: 5′GAGGTGGATTCCGCTCCCCA3′
CK-19R: 5′CTGCTCGAGGGACAGGAAGAT3′
CK-19P: CCTGGTTCACCAGCCGGACTGAAGAATTGAACCGGG

Mammaglobin B (corresponding gene is *SCGB2A1*):

MGB-2F: 5′ACTCCTGGAGGACATGGTTGA3′
MGB-2R: 5′TCTGAGCCAAACGCCTTGGGT3′
MGB-2P: GTGATGCCGCTGCAGAGGCTCTGGGGAAATTCAAGC

NY-BR-1 (corresponding gene is *ANKRD30A*):

NYBR1A: 5′CAAAGCAGAGCCTCCCGAGAAG3′
NYBR1B: 5′CCTATGCTGCTCTTCGATTCTTCC3′

To confirm the synthesis of cDNA, all samples were amplified with a set of primers resulting in a 542 bp fragment of the RNF38 gene:

Housekeeping gene RNF38 (corresponding gene is *RNF38*):

RNF38F: 5′AAAAAGTCTTTGGAGTTCCATCAC3′
RNF38R: 5′GAAGATGGAGAAGTAGAAAATTAC3′

### DNA amplification

Independent amplification reactions were performed with the different primer sets. Cytokeratin-19 and NY-BR-1 were amplified in a 50 *μ*l reaction mixture containing 5 pmol of both primers, 2 mM MgCl_2_, 200 *μ*mol l^−1^ of each deoxyribonucleoside triphosphate, and 2.5 U of the recombinant FastStart Taq DNA Polymerase in 1 × GC-RICH solution (Roche, Mannheim, Germany). Negative and positive controls were amplified in every experiment, as well as samples devoid of DNA. Polymerase chain reaction was performed in a DNA thermal cycler (MJ Research, Watertown, MA, USA). To ensure full denaturation, the mixtures were heated at 94°C for 4 min, followed by 40 and 30 amplification cycles, respectively, of 45 s at 94°C, 40 s at 58°C, and 45 s at 72°C. MGB-2 was amplified in the same reaction mixture for 40 cycles at the same conditions except for annealing carried out at 55°C for 30 s and extension at 72°C for 1 min. RNF38 was amplified with Amplitaq polymerase (Roche, Mannheim, Germany) for 30 cycles at 94°C for 45 s, 52°C for 30 s and 72°C for 1 min. An additional 5 min elongation at 72°C was included for all amplifications. For analysis of PCR products 10 *μ*l of the amplification products were electrophoresed on a 2% agarose gel (BMA, Rockland, MA, USA) and visualised by ultraviolet illumination after ethidium bromide staining.

### Southern blot

Southern blot of the PCR products was performed on a Genescreen Plus membrane in 20 × SSC. Hybridisation was performed in Rapid-hyb buffer (Amershampharmacia biotech, Piscataway, NJ, USA) with oligonucleotide probes (CK-19P and MGB-2P) previously biotin labelled by biotin N^6^-ddATP (10 pmol ml^−1^, NEN Life Science Products Inc., Boston, MA, USA). Detection was carried out following incubation with streptavidin–HRP conjugate and visualised by chemiluminescence (NEN Life Science Products Inc., Boston, MA, USA) according to the instructions of the manufacturer.

### Data analysis

Summary statistics were obtained using established methods. Differences between categorical variables were calculated using either *χ*^2^ test or Fisher's exact test as appropriate. A *P*-value <0.05 was considered to be significant. Statistical analysis was performed with SPSS® version 10 for Windows statistical package (SPSS Inc., Chicago, IL, USA).

## RESULTS

In all, 30 SLNs from 28 patients were studied. RNA was extracted and cDNA synthesised successfully from all 30 SLNs. The presence of cDNA in all samples was confirmed by PCR with a set of primers which amplifies a 542 bp segment of the RNF38 ubiquitously expressed gene (data not shown).

**Figure 1:**
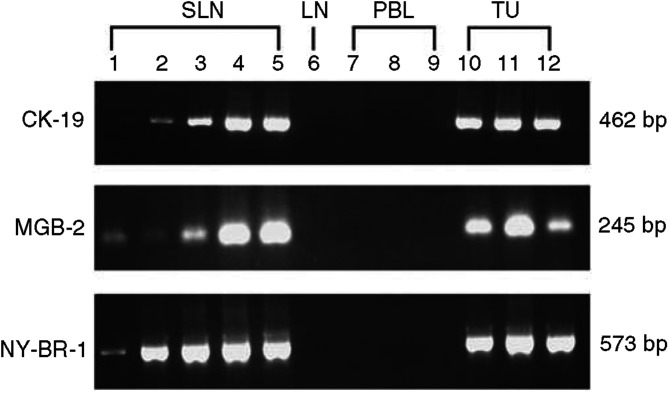
Gel electrophoresis of PCR products of the three molecular markers. Five representative sentinel lymph nodes (SLN) were chosen and amplified again for demonstration of the three markers used in the study: cytokeratin-19 (CK-19), mammaglobin-2 (MGB-2) and NY-BR-1. All five SLNs showed amplification of all three markers (lanes #1–5). Representative negative controls including one lymph node obtained from a patient with disease other than breast cancer (LN, lane #6) and peripheral blood lymphocytes obtained form healthy volunteers (PBL, lanes #7–9). Three primary tumours were used as positive controls (TU, lanes #10–12).

Enhanced pathologic analysis by H&E detected breast cancer metastasis in 6/30 nodes (20.0%) and IHC for CK was positive in the same six nodes and in two additional lymph node (8/30, 26.7%). Reverse transcriptase–polymerase chain reaction for at least one of the three markers was positive in 15/30 nodes (50.0%) including all nodes positive by IHC and seven additional nodes. These results are outlined in Table 1 and Figure 1.

The sensitivity of IHC for CK in the detection of breast cancer micrometastases in SLNs was increased by 6.7% as compared to the H&E stain alone. Reverse transcriptase–polymerase chain reaction increased sensitivity in additional 23.3% compared to combined H&E and IHC stains and by 30.0% as compared to H&E alone (Table 2).

All three markers were highly expressed in 5/6 (83.3%) H&E-positive SLNs. In one patient where SLN was positive both by H&E and IHC, only MGB-2 and NY-BR-1 were expressed while RT–PCR for CK-19 was negative (case # 28, Table 1).

Reverse transcriptase–polymerase chain reaction was positive for all three markers in 7/15 (46.7%) of positive SLNs, for two markers in 3/15 (20%) of positive SLNs, and for a single marker in 5/15 (33.3%) of positive SLNs.

Reverse transcriptase–polymerase chain reaction was positive for CK-19, in 8/30 (26.7%) SLNs, for MGB-2, in 11/30 (36.6%) SLNs, and for NY-BR-1, in 13/30 (43.3%) patients (Table 3).

Three primary tumours of breast cancer patients were used as positive controls. All three tumours showed strong amplification for all three markers (Figure 1). Lymph nodes of three patients with benign lymphadenopathy biopsied for diagnostic purposes (reactive lymph nodes by histology without evidence of malignancy) and peripheral blood lymphocytes of 10 healthy volunteers were used as negative controls. None of the 13 negative control specimens were amplified by any of the three RT–PCR markers used in the study.

### Southern blot

In order to verify the RT–PCR results, Southern blot was performed for CK-19 and MGB-2. A probe for NY-BR-1 was constructed but failed to show any significant staining, therefore we elected to confirm the RT–PCR results by CK-19 and MGB-2 only. The results are outlined in Table 1. There was no difference between the Southern blot results and the RT–PCR results for both markers.

## DISCUSSION

Diagnosis and staging of malignant diseases using molecular biology techniques focused initially on the analysis of the primary tumour. Despite large amount of data showing relationship between molecular findings and patient's outcome, very few molecular biology techniques are being used in routine clinical practice. The search for MRD in sites other than the primary tumour such as lymph nodes, bone marrow or peripheral blood is a different direction of utilising sensitive molecular biology techniques for cancer staging and improving treatment selection.

The ideal molecule for MRD detection would be a molecule expressed in all tumour cells but not in normal human tissues. Such molecules are rare and most of the investigators focus on molecules expressed in tumour and normal tissues but not expressed in the target organ. Such an example is the utilisation of tyrosinase, an enzyme present only in melanocytes, for the detection of MRD in melanoma patients (Li *et al*, 2000). In breast cancer patients, molecules such as CK, expressed in epithelial cells but not expressed in lymphocytes, were investigated (Kruger *et al*, 1996; Manhani *et al*, 2001; Ismail *et al*, 2003). Using PCR to detect nonspecific epithelial markers may yield a robust and simple assay for the detection of MRD in patients with epithelial-derived tumours. However, the risk of false-positive results due to contamination by epithelial cells during the surgical procedure is high. On the other hand, tumour-specific molecular markers are not uniformly expressed in all tumours and therefore if tumour tissue is not available for analysis or the selected marker is not expressed, the assay will be inconclusive.

In the present study, we elected to combine a nonspecific epithelial marker (CK-19) with breast cancer-specific markers (MGB, NY-BR-1) in order to achieve high sensitivity in MRD detection.

Our results show that the addition of immunohistochmical staining of the SLN for CK increased the SLN positivity rate from 20 to 26.7%. This is well within the range reported in the literature (Euhus, 2003; Chao, 2004). Staging by RT–PCR increased the number of positive SLN from 20 to 50% when any positive marker is considered. All three molecular markers were positive in all three primary tumour tissues and negative in all 13 negative controls. However, if only SLNs with all three molecular markers were considered positive we would have missed one of the six H&E-positive SLNs and one of the two IHC-only-positive SLNs. One additional SLN would have been considered positive. When any two molecular markers were considered positive, all H&E- and IHC-positive SLNs with additional two SLNs would have been positive by RT–PCR.

A single molecular marker was positive in 50% of the SLNs, the significance of a positive single molecular marker is yet to be determined. The most sensitive molecular marker was NY-BR-1 being positive in 13 SLNs, followed by MGB-2 in 12 SLNs and CK-19 in eight. Interestingly, the two specific markers were more sensitive compared to the epithelial marker (CK-19), which is expected to yield a certain degree of false-positive results due to contamination by epithelial cells at the time of surgery. This can be explained by the surgical technique used in the present study where careful attention was paid not to use contaminated instruments to handle the SLN. Mammaglobin was studied before by other investigators, (Ouellette *et al*, 2004) who examined the expression of MGB-1and -2 in 22 SLNs from breast cancer patients. They found a sensitivity of 88% and specificity of 72% as compared to the histological status. Other investigators (Ooka *et al*, 2000) studied several markers in non-SLNs from 17 breast cancer patients. In their study, prostate-specific antigen (PSA), carcinoembryonic antigen (CEA), and MGB-1 and -2 were highly expressed in the primary tumours. Mammaglobin-1 and -2 were 100% positive in the H&E-positive lymph nodes while CEA and PSA were positive in 35.7 and 57.1% of the H&E-positive lymph nodes. Mammaglobin-1 and -2 were amplified in additional 30.1 and 17.8% of the H&E-negative nodes. In an interim analysis of a multi-institutional study of multimarker RT–PCR of H&E-negative lymph nodes (Gillanders *et al*, 2004), a correlation between PCR positivity and other well-established prognostic markers including histological grade and St Gallen risk category was reported. This study also showed mammaglobin to be the most informative marker in their multimarker panel.

Our results with MGB-2 are close to results reported by others. Our results show that multimarker RT–PCR is more sensitive as compared to the standard pathologic processing of the SLN by serial sections and IHC and that a multimarker approach is much more sensitive as compared to any single marker. NY-BR-1 was more sensitive than MGB-2 and the only molecular marker positive in all H&E- and IHC-positive nodes. Despite its high sensitivity, NY-BR-1 is not uniformly expressed in all breast cancers and since fresh primary tumour tissue is not always available for molecular analysis, it could not be used as a single marker. In the future, when anti-NY-BR-1 antibodies will be available, its expression on the primary tumour cells could be determined by IHC and when expressed, it can be used as a single molecular marker in the NY-BR-1-positive tumours. Until then, a multimarker assay would be the best choice for the determination of MRD in node-negative breast cancer patients. The significance of MRD detected by RT–PCR in SLNs of breast cancer patients is yet to be determined. The current AJCC-UICC staging system defines micrometastasis as tumour deposits of <2 mm in diameter and cell clusters as tumour cells within the lymph node with a diameter of <0.2 mm, regardless of the staining system used for their detection (Singletary *et al*, 2003). Controversy exists not only regarding the management of breast cancer patients with micrometastasis but also regarding patients with macrometastasis detected in the SLN. The management of breast cancer patients with macroscopic SLN metastasis is currently investigated in two prospective randomised clinical trials, the EORTC 10981-22023 (AMAROS) trial and the ACOSOG Z011(Mansel and Goyal, 2004; Posther *et al*, 2004). Both trials comparing axillary lymph node dissection to radiation therapy (AMAROS) and to no further surgery (Z011) in T1–T2 breast cancer patients with positive SLN. Two other clinical trials, the IBCSG 23-01 trial and the ACOSOG Z010 (Mansel and Goyal, 2004; Posther *et al*, 2004) are studying the significance of micrometastasis detected by either IHC or H&E in SLN of breast cancer patients. The significance of RT–PCR in molecular staging of breast cancer will be determined not only by these ongoing clinical trials but also by future large-scale prospective trials stratifying patients according to the volume of metastatic disease in the SLN.

## Figures and Tables

**Table 1:** Analysis of sentinel lymph nodes for the presence of breast cancer metastases by H&E, IHC and RT–PCR

			**CK-19**		**NY-BR-1**		**MGB-2**	
	**H&E**	**IHC**	**RT**–**PCR**	**SB**	**RT**–**PCR**	**SB^a^**	**RT**–**PCR**	**SB**
1	−	+	+	a	+		+	a
2	−	+	+	a	+		−	a
3	−	−	+	+	+		++	+++
4	−	−	−	−	−		−	−
5	−	−	−	−	−		−	−
6	−	−	−	−	+		−	−
7	+	+	+	+	+		+	+
8	+	+	+++	+	+++		+++	+
9	−	−	−	−	−		+	+
10	−	−	−	−	−		−	−
11	−	−	−	−	−		−	−
12	−	−	−	−	−		−	−
13	−	−	−	−	−		−	−
14	−	−	−	−	−		−	−
15	−	−	−	−	−		+	+
16	−	−	−	−	−		−	−
17	−	−	−	−	+		−	−
18	−	−	−	−	+		−	−
19	−	−	−	−	−		−	−
20	−	−	−	−	−		−	−
21	−	−	−	−	++		++	++
22	+	+	+	+	+		++	+
23	−	−	−	−	−		−	−
24	−	−	−	−	−		−	−
25	−	−	−	−	−		−	−
26	+	+	+++	+	+++		+++	+
27	+	+	+++	+	+++		+++	+
28	+	+	−	−	+		++	+
29	−	−	−	−	−		−	−
30	−	−	−	−	−		−	−

a Southern blot was not performed due to technical reasons.

H&E=haematoxilyn and eosin stain; IHC=immunohistochemistry stain for cytokertain; RT–PCR=reverse transcriptase–polymerase chain reaction; CK-19=cytokeratin-19; MGB-2=mammaglobin-2; SB=southern blot.

**Table 2:** Comparison of different detection methods for breast cancer sentinel lymph-node metastases

**Method**	**Total positive**	**% Positive^a^**	**Absolute sensitivity increase (%)**	**Relative sensitivity increase^b^ (%)**
H&E	6	20.0	0	0
IHC	8	26.7	6.7	33.3
RT–PCR	15	50.0	30.0	150.0

a Percentage of positive nodes out of all nodes analysed (*N*=30).

b Percentage increase in positive nodes detected using number of nodes detected by H&E as a baseline (*N*=6).

H&E=haematoxilyn and eosin stain; IHC=immunohistochemistry stain for cytokertain; RT–PCR=reverse transcriptase–polymerase chain reaction.

**Table 3:** Increment sensitivity of SLN analysis for breast cancer metastases

	**Frequency of positive SLN**	**Percentage of positive SLN (%)**	***P*-value** *
H&E	6	20	
IHC	8	26.7	<0.01
NY-BR-1	13	43.3	<0.01
CK-19	8	26.7	<0.01
MGB-2	11	36.7	<0.01
3 markers	7	23.3	<0.01
2 markers	10	33.3	<0.01
1 marker	15	50.0	<0.01

* Fisher's exact test two-tailed *P*-value (compared to H&E alone).

SLN=sentinel lymph node; H&E=haematoxylin and eosin stain; IHC=immunohistochemistry stain for cytokeratin.
